# Seroprevalence of *Toxoplasma gondii* Infection in Patients of Intensive Care Unit in China: A Hospital Based Study

**DOI:** 10.1155/2015/908217

**Published:** 2015-04-16

**Authors:** Yong-Biao Zhang, Wei Cong, Zhi-Tao Li, Xiao-Gang Bi, Ying Xian, Yan-Hong Wang, Xing-Quan Zhu, Kou-Xing Zhang

**Affiliations:** ^1^Department of Intensive Care Unit, The Third Affiliated Hospital, Sun Yat-sen University, Guangzhou, Guangdong 510630, China; ^2^State Key Laboratory of Veterinary Etiological Biology, Lanzhou Veterinary Research Institute, Chinese Academy of Agricultural Sciences, Lanzhou, Gansu 730046, China; ^3^College of Animal Science and Technology, Jilin Agricultural University, Changchun, Jilin 130118, China; ^4^Jiangsu Co-Innovation Center for the Prevention and Control of Important Animal Infectious Diseases and Zoonoses, Yangzhou University College of Veterinary Medicine, Yangzhou, Jiangsu 225009, China

## Abstract

The objective of this study was to estimate the seroprevalence of* Toxoplasma gondii* infection in 394 patients of intensive care unit (ICU) in a hospital between April 2010 and March 2012 and analyze the association between* T. gondii* infection and ICU patients according to the species of disease.* Toxoplasma* serology was evaluated by ELISA method using a commercially available kit. Data of patients were obtained from the patients, informants, and medical examination records. Seventy-four (18.78%) of 394 patients were positive for anti-*T. gondii* IgG antibodies demonstrating latent infection. Of these, the highest* T. gondii *seroprevalence was found in the age group of 31–45 years (27.45%), and the lowest was found in the age group of <30 years (12.5%). In addition, females (21.6%) had a higher seroprevalence than males (18.36%). With respect to the species of disease, the patients with kidney diseases (57.14%), lung diseases (27.84%), and brain diseases (24%) had high* T. gondii* seroprevalence. The present study represents the first survey of* T. gondii* seroprevalence in ICU patients in China, revealing an 18.78% seropositivity. Considering the particularities of ICU patients, molecular identification, genetic characterization, and diagnosis of* T. gondii* should be considered in future study.

## 1. Introduction

Toxoplasmosis, a zoonotic infection of humans and animals, is caused by the ubiquitous obligatory intracellular coccidian protozoan* Toxoplasma gondii*, which is an opportunistic parasitic infection in immune-compromised hosts worldwide, and approximately one-third of the global population has been computed to be carrying the parasite [1]. Human infection with* T. gondii* has been reported in China, with a mean infection rate of 7.9% nationwide, as revealed by ELISA between 2001 and 2004 [2]. However, in recent years, a large amount of epidemiological investigation experiments have been conducted in various groups, including normal population, pregnant women, cancer patients, and psychiatric patients, indicating that* T. gondii* infection is actually a significant human health problem in China [3–6].* T. gondii* infections in humans are usually acquired through ingesting tissue cysts of the parasite in raw or undercooked meat, by ingesting parasite oocysts in feline faeces that contaminate drinking water, soil, vegetables, and other food sources, and transplacentally from infected mothers to their infants [1, 7].

In immunocompetent individuals, most* T. gondii *infections are asymptomatic. Nevertheless, in immunocompromised hosts, an increased risk of reactivation of latent infection in various organs may occur, resulting in severe diseases or even death [8]. Patients in the ICU are usually considered to be particularly immunocompromised; they may be extremely susceptible to the reactivation of* T. gondii* infection. Thus, detection and surveillance of anti-*Toxoplasma* antibodies are of great interest, especially in ICU patients presenting with at least one form of organ failure [9, 10]. However, epidemiological knowledge regarding the prevalence of* T. gondii* infection in ICU patients is unavailable in China. Therefore, the present study was conducted to estimate the seroprevalence of* T. gondii* infection in ICU patients in China for the first time, aiming to evaluate the risk for reactivation of* T. gondii *infection in ICU patients in China.

## 2. Methods and Materials

### 2.1. Ethics Statement

This study was approved before its commencement by the Ethics Committee of the Third Affiliated Hospital, Sun Yat-sen University. The sera were collected with agreement from the volunteers and guardians.

### 2.2. Serum Samples and Serological Examination

Three hundred and ninety-four (207 male, 125 female, and 62 no data) ICU patients who presented to the Third Affiliated Hospital, Sun Yat-sen University, Guangdong province, China, between April 2010 and March 2012 were included in the study. Each patient required intensive care management, as they displayed at least one form of organ failure. Blood sample was taken from all the ICU patients under sterile conditions, and the sera were separated and stored at −20°C until further testing. In addition, data of patients were obtained from the patients, informants, and medical examination records, including age, gender, and species of disease. All the serum samples were tested for* T. gondii* IgG antibodies by enzyme-linked immunosorbent assay (ELISA) using a commercially available kit (Haitai Co., Ltd., China) according to the manufacturer's instructions. Positive and negative serum controls were included in every plate. All samples were run in triplicate.

### 2.3. Statistical Analyses

All data were processed and analyzed by SPSS 19.0 Data Editor (SPSS Inc., Chicago, Illinois, USA). *χ* ^2^-test and Kolmogorov-Smirnov one-sample test were used. All statistical tests were two-sided. The results in comparison with groups were considered different if *P* < 0.05.

## 3. Results 

Seventy-four (18.78%, 95% CI: 14.93%–22.64%) of 394 ICU patients were positive for anti-*T. gondii* IgG antibodies demonstrating latent infection with* T. gondii.* Of these, the highest seroprevalence of* T. gondii *infection was found in the age group of 31–45 years (27.45%) and the lowest was found in the age group of <30 years (12.50%). In addition, females (21.60%) had a higher seroprevalence than males (18.36%), but the difference was not significantly different (*P* = 0.47) (Table 1).

The seroprevalence of* T. gondii* infection in the patients with respect to the species of disease is shown in Table 2, and the highest* T. gondii* seroprevalence was found in patients with kidney diseases (57.14%), which is significantly higher than others. In addition, high prevalence of latent* T. gondii* infection was also found in patients with lung diseases (27.84%, 95% CI: 18.92%–36.75%) and brain diseases (24.00%, 95% CI: 12.16%–35.84%).

## 4. Discussion

*T. gondii* infection in humans is common all over the world, with the prevalence varying in accordance with environment, eating habits, and age. In the present study, we found an 18.78% seroprevalence of* T. gondii* in ICU patients, which is significantly higher than that in people who lived in Meixian (10.12%) in Guangdong province [11], and 7.50%* T. gondii* seroprevalence in blood donors in Guangdong province [12]. This difference suggests that ICU patients may be more susceptible to* T. gondii* infection. Therefore, any immunocompromised patients should be requested for the detection of* T. gondii* infection, and those who are at high risk of being infected should be considered carefully. Moreover, in Guangdong province, wildlife is very popular in their daily diet and it is common to eat dogs and cats for local population. All of these may contribute to the acquisition of* T. gondii* infection.

Toxoplasmosis was considered to be acquired in the early stage and the prevalence is enhanced with age and declines in later stage [10, 13]. From the present study, higher seroprevalence of* T. gondii* infection was found in younger population compared to older age group, which was consistent with the abovementioned studies. Toxoplasmosis was considered as an important infectious syndrome, and the main clinical feature is the enlargement of the spleen, the liver, and/or the lymph nodes and it can also influence other organs such as the heart, central nervous system (CNS), or eyes [14]. In the present study, high seroprevalence was found in patients with kidney disease, lung disease, and brain disease, and most of the patients revealed at least one form of organ failure. However, it can not be ignored that the sample size for kidney disease is so small; thus, this warrants further large-scale case-control studies to confirm the present result.

Usually, diagnosis of* T. gondii* infection can be divided into two categories: direct methods and indirect methods. Direct methods include polymerase chain reaction (PCR), hybridization, isolation, and histology. Indirect methods mainly involve serological methods [15]. In our patients, serology was useful. In immunocompetent patients, indirect serological methods are extensively used because they are faster and cheaper than other methods. However, in asymptomatic patients who are immunocompromised, testing for IgG antibodies to* T. gondii* should also be executed, as this allows us to identify who are at risk for the reactivation of latent infection [16]. Moreover, detection of IgM antibodies is useful to indicate newly acquired* T. gondii* infection, but the possibility of false IgM positive results should not be discarded.

In immunocompromised patients, direct methods for detecting* T. gondii* infection must be employed. PCR amplification of* T. gondii* genes (particularly, the* B1* gene) should be employed to detect* T. gondii* in body fluids and tissues of patients [15]. Acute infection could be indicated by directly isolating* T. gondii* from blood or body fluids, whether newly acquired or reactivation of latent infection. In addition, tissue sections or body fluid smears could also be used to observe tachyzoites.

In conclusion, the present study revealed for the first time the seroprevalence of* T. gondii* infection in ICU patients in Guangdong province, China. However, some defects are present in the present study: the data represented only a specific province of China and the numbers of patients in the different age groups are small. Further studies should be conducted to estimate the prevalence of* T. gondii* infection in ICU patients in other provinces of China and increase the size of patients in the different age groups. Second, infection status (current infection or past infection) could not be clearly displayed by the serology, and potential bias could not be excluded due to misclassification. Third, considering association analysis, cross-sectional study design has its shortcoming. Therefore, further large-scale case-control studies or prospective studies should be conducted to confirm our findings.

## Figures and Tables

**Table 1:** Seroprevalence of *Toxoplasma gondii *infection in 394 intensive care unit patients in China.

Characteristic	Number of subjects tested	Prevalence (%) (95% CI)	*P* value
Age groups (years)			
30 or less	40	12.50 (2.25–22.75)	
31–45	51	27.45 (15.20–39.70)	0.10
46–60	64	20.31 (10.47–30.17)	0.31
61–75	95	20.00 (11.96–28.04)	0.30
>75	82	17.07 (8.93–25.22)	0.51
No data	62	14.52 (5.75–23.29)	0.77
Gender			
Male	207	18.36 (13.08–23.63)	
Female	125	21.60 (14.39–28.81)	0.47
No data	62	14.52 (5.75–23.29)	0.48

**Table 2:** *Toxoplasma gondii* infection in 394 intensive care unit patients with different species of disease.

Species of disease	Number of subjects tested	Prevalence (%) (95% CI)
Liver disease	31	19.36 (5.45–33.26)
Lung disease	97	27.84 (18.92–36.75)
Kidney disease	7	57.14 (20.48–93.80)
Heart disease	95	11.58 (5.15–18.01)
Brain disease	50	24.00 (12.16–35.84)
Other diseases	114	12.28 (6.26–18.31)
Total	394	18.78 (14.93–22.64)
